# Low light visual function after accelerated corneal Cross-Linking Protocols: 18 mW/cm2 vs. 9 mW/cm2

**Published:** 2018

**Authors:** Soheila Asgari, Hassan Hashemi, Ebrahim Jafarzadehpur, Alireza Mohamadi, Shiva Mehravaran, Akbar Fotouhi

**Affiliations:** * Department of Epidemiology and Biostatistics, School of Public Health, Tehran University of Medical Sciences, Tehran, Iran; ** Noor Ophthalmology Research Center, Noor Eye Hospital, Tehran, Iran; *** Noor Research Center for Ophthalmic Epidemiology, Noor Eye Hospital, Tehran, Iran; **** Department of Optometry, School of Rehabilitation Sciences, Iran University of Medical Sciences, Tehran, Iran; ***** ASCEND Center for Biomedical Research, Morgan State University, Baltimore, MD, USA

**Keywords:** mesopic, scotopic, visual function, cross-linking, comparative study

## Abstract

**Objective.** To compare one-year results of vision, corneal aberrometry and contrast sensitivity (CS) in low light conditions between 5- and 10-minute accelerated cross-linking (CXL) protocols.

**Methods.** Thirty eyes were evaluated in each studied group. Uncorrected (UDVA) and corrected (CDVA) distance visual acuity by the SC-2000 Snellen chart, corneal higher order aberrations using the OPD Scan III and CS using MonCv3System was tested under mesopic (20 lux) and scotopic (0.5 lux) light conditions at pre-CXL and 6 and 12 months post-CXL.

**Results.** At 12 months, a mean improvement of 0.06±0.22 (22.2%) and 0.02±0.25 logMAR (7.9%) in mesopic UDVA and 0.01±0.13 (14.3%) and 0.07±0.13 logMAR (87.9%) in mesopic CDVA was observed in the 5- and 10-minute groups, respectively. Mean decline in scotopic UDVA was 0.01±0.16 (1.0%) and 0.03±0.17 logMAR (11.9%) and mean improvement in scotopic CDVA was 0.03±0.10 (35.5%) and 0.02±0.07 logMAR (22.2%), respectively. Inter-group differences in the decrease of corneal aberrations were not statistically significant. Among CS variables, only inter-group changes in corrected CS 0.5 to 2.2 was significantly different (all P<0.050). The linear regression analysis showed that these differences were related to baseline values not CXL protocols; corrected CS 0.5 (Pgroup=0.261 and Pbaseline value<0.001), CS 1.1 (Pgroup=0.250 and Pbaseline value<0.001), and CS 2.2 (Pgroup=0.101 and Pbaseline value=0.054).

**Conclusions.** Changing the intensity of UV in cross-linking from 18mW/ cm2 to 9mW/ cm2 does not affect the visual function under low-light conditions.

## Introduction

Keratoconus leads to progressive irregular astigmatism and deteriorating visual acuity. Corneal cross-linking (CXL) with standard and different accelerated protocols contributes to ectasia stabilization, corneal flattening, and reduction of irregular astigmatism by increasing collagen cross-links and corneal strengthening [**1**]. 

Today, accelerated protocols, which reduce the irradiation time while maintaining the total power, are receiving high consideration. Different studies have presented the safety and effectiveness of these protocols. Commonly studied protocols are the 9mW/ cm2 for 10 minutes and the 18mW/ cm2 for 5 minutes. In comparative studies and randomized trials, short [**2**] and long term [**3**] results of these accelerated protocols have been compared to the standard method. For example, a one-year study of the 10-minute versus the standard method [**4**] showed a comparable significant enhancement of visual acuity and decline of refraction with the two methods. However, there was more reduction in cylinder refraction in the standard group than in the 10-minute group. In an 18-month RCT [**3**], we observed similar vision and refraction results with the standard and 5-minute methods, but there was better corneal flattening with the standard method. One study also compared various accelerated methods versus the standard and showed better vision improvement with the 5-minute protocol than the 10-minute one [**5**]. In a 2011 study on 70 keratoconus eyes by this team [**6**], we demonstrated reduced corneal higher order aberrations (HOAs) following the 5-minute protocol. 

Since vision problems in keratoconus patients are exacerbated in low light conditions and with pupil dilation [**7**], in this study, we compared the 5- and 10-minute CXL protocols regarding vision, aberrations, and contrast sensitivity in low light, including mesopic (20 lux) and scotopic (0.5 lux) conditions to reach an accurate judgment of their effects.

## Materials and methods

This report is part of a non-randomized clinical trial of progressive keratoconus patients in Noor Eye Hospital since June 2013. Enrolled patients were assigned either to the 5-minute (30 eyes from 22 cases) or the 10-minute CXL (30 eyes from 24 cases) group. Inclusion criteria of this report were diagnosis of progressive keratoconus (increasing one diopter or more in maximum simulated keratometry, refractive cylinder, or manifest refraction spherical equivalent or the decline of at least 2 lines of corrected distance visual acuity (CDVA) during the past 12 months), age range 15 to 35 years, maximum keratometry (Kmax) less than 55.0 diopters (D), and thinnest corneal thickness of 400μm. The grading was done using the Pentacam (Oculus Optikgerate GmbH, Wetzlar, Germany) based on an index of surface variance (ISV) of 30-90 and a keratoconus index (KI) of 1.07 to 1.25 [**8**]. Cases with any past ophthalmic surgery or other eye diseases were not included. Contact lenses users discontinued using them before examinations and surgery; the minimum interval for hard and soft lenses was 3 weeks and 3 days, respectively. 

The Ethics Committee of Tehran University of Medical Sciences accredited the study protocol. The study adhered to the Declaration of Helsinki at all stages. Written informed consents were obtained from all study participants. 

## Surgical technique

Proparacaine hydrochloride 0.5% was used for local anesthesia. The lid speculum was removed after removing the epithelium of the central 9.0mm cornea. Then, instillation of riboflavin solution (Streuli Pharma, Uznach, Switzerland) was repeated every 3 minutes for 30 minutes. An irradiance of 18mW/ cm2 was applied in the 5-minute group, and of 9mW/ cm2 in the 10-minute group. After rinsing the corneal surface and placing a bandage lens (Night & Day, Ciba Vision, Duluth, USA), levaquin eye drops were used. Postoperative medical regiment was levofloxacin and betamethasone 0.1% every 6 hours and artificial tears as needed. The bandage lens was withdrawn after re-epithelialization was observed in daily postoperative examinations. Then, levaquin was terminated and betamethasone was prescribed 4 times a day for one week.

## Pre-and post-CXL examinations

Uncorrected (UDVA) and corrected (CDVA) distance visual acuity were tested by the Snellen chart SC-2000 (Nidek Inc., Tokyo, Japan) with black background letters chart under mesopic and scotopic conditions. Also, corneal higher order aberrations (HOAs) were evaluated by the mesopic setting with OPD-Scan lll (Nidek Inc., Tokyo, Japan) and scotopic contrast sensitivity at SP of 0.5, 1.1, 2.2, 3.4, 7.1, and 15 cpd using MonCv3System (Metrovision, France) at baseline, and at 6 and 12 months after CXL. The same optometrist completed all tests pre- and post-CXL in the study room. 

To adjust light, the test room was dark completely, and an ambient light source provided the lighting condition. Light was measured using the Sekonic L-308DC (Sekonic Corporation, Tokyo, Japan). Illuminance was set at 20 lux for mesopic condition and 0.5 lux for scotopic situation. Patients stayed in the room 10-15 minutes to adapt to each light condition. Pupil diameter was 6.86±0.08 and 6.75±0.21 mm in 5 min and 10 min-group, respectively (P=0.625).

## Statistical analyses

One-year changes of the studied indices was compared between the 5- and 10-minute groups using analysis of variance by adjusting for the correlation between two eyes in bilateral cases and baseline value of indices. Safety index was determined as postoperative CDVA/ preoperative CDVA, and independent sample t test was used to compare two groups. The linear regression model showed the concurrent effect of baseline value and study groups on changing the indices. 

## Results

In the 5- and 10-minute groups, the mean age was 24.3 ±5.2 and 22.4 ±6.0 years (P=0.271), and the percentage of male patients was 53.1% and 56.0%, respectively. At baseline, K-max was 46.6 ±2.2 and 47.6 ±2.1D (P=0.162) and K-min was 43.7 ±1.5 and 43.7 ±1.9D (P=0.929) in the 5- and 10-minute groups, respectively.

## Inter-group comparisons of baseline data

**Fig. 1:**
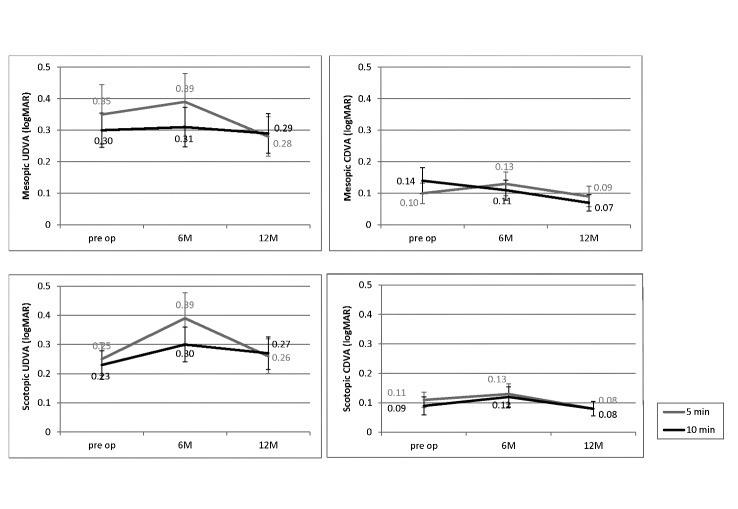
Comparison of mean uncorrected distance visual acuity (UDVA) and corrected distance visual acuity (CDVA) in mesopic and scotopic conditions between 5-minute and 10-minutes cross-linking protocols

Inter-group differences of baseline mesopic and scotopic UDVA and CDVA (**Fig. 1**), mesopic HOAs (**Table 1**), and scotopic corrected and uncorrected contrast sensitivity (all P>0.050) were non-significant, except for corrected contrast 0.5 (P=0.037), 1.1 (P=0.023) and 2.2 (P=0.051) (**Table 2**,**3**). 

**Table 1:** Comparison of mesopic corneal higher order aberrations between 5-minute and 10-minute cross-linking protocols

		Pre-operative	Follow up		One-year change	P-value
			6 months	12 months		
Total HOA	5 min	3.80±1.97	3.25±1.74	3.33±1.68	-0.47±1.90	0.653
	10-min	3.89±5.43	2.80±1.58	2.86±1.70	-0.99±3.86	
coma	5-min	3.10±1.59	2.78±1.80	2.84±1.64	-0.26±1.09	0.634
	10-min	2.92±3.50	2.17±1.39	2.29±1.58	-0.62±3.19	
Trefoil	5-min	1.23±0.89	0.95±0.40	1.06±0.45	-0.18±0.96	0.867
	10-min	1.43±1.76	1.13±0.78	1.17±0.89	-0.24±1.18	
Spherical aberration	5-min	1.90±5.06	0.61±0.36	0.72±0.61	-1.17±5.06	0.463
	10-min	0.86±1.77	0.58±0.61	0.51±0.39	-0.33±1.62	

**Table 2:** Comparison of scotopic corrected contrast sensitivity (CS) between 5-minute and 10-minute cross-linking protocols

	Study group	Baseline	Follow up		12 month change	P-value
			6 months	12 months		
CS0.5 (dB)	5 min	14.91±3.30	16.35±2.35	17.00±1.86	2.04±3.96	0.031
	10-min	12.60±5.03	14.00±2.55	18.40±2.79	5.00±5.66	
CS1.1 (dB)	5-min	17.61±3.56	18.83±2.19	19.70±2.64	2.04±4.36	0.015
	10-min	14.60±6.91	17.60±1.14	21.20±3.11	5.67±7.47	
CS2.2 (dB)	5-min	19.30±1.69	19.83±2.77	20.39±3.01	1.21±20.59	0.016
	10-min	19.00±1.22	19.00±0.50	22.20±3.35	3.33±2.50	
CS3.4 (dB)	5-min	18.48±2.48	18.70±2.36	19.83±2.87	1.29±2.29	0.139
	10-min	18.80±2.39	18.80±0.84	21.80±3.56	2.50±2.07	
CS7.1 (dB)	5-min	14.87±3.39	16.48±2.91	16.22±4.19	1.45±4.31	0.110
	10-min	14.60±3.97	15.20±0.84	19.00±4.30	4.33±0.82	
CS15 (dB)	5-min	7.39±3.54	8.30±2.91	8.65±4.30	1.12±4.63	0.414
	10-min	7.80±4.87	7.40±2.88	11.20±4.09	2.50±3.02	

**Table 3:** Comparison of scotopic uncorrected contrast sensitivity (CS) between 5-minute and 10-minute cross-linking protocols

	Study group	Baseline	Follow up		12 month change	P-value
			6 months	12 months		
CS0.5 (dB)	5 min	15.64±1.55	16.07±1.21	16.50±2.77	0.87±1.60	0.259
	10-min	15.00±1.41	15.00±1.40	16.75±3.95	1.60±2.61	
CS1.1 (dB)	5-min	16.93±3.43	17.29±2.76	18.07±3.15	0.87±2.53	0.364
	10-min	16.00±5.89	15.00±3.16	19.00±4.24	1.80±3.56	
CS2.2 (dB)	5-min	16.50±3.13	16.64±2.98	18.36±3.85	1.43±3.07	0.978
	10-min	15.75±4.27	13.50±1.29	18.25±6.55	1.40±4.83	
CS3.4 (dB)	5-min	15.43±4.60	15.21±3.47	16.93±4.71	0.48±3.96	0.487
	10-min	15.00±7.70	13.50±3.32	16.75±6.18	1.60±2.61	
CS7.1 (dB)	5-min	13.00±5.70	13.86±4.26	14.21±6.45	0.04±3.73	0.143
	10-min	13.00±8.91	10.50±4.65	15.75±9.36	1.80±3.96	
CS15 (dB)	5-min	5.14±3.39	5.43±3.84	7.14±5.71	0.61±4.27	0.258
		4.75±5.25	4.00±4.62	8.00±9.27	2.20±5.85	

## Intergroup comparisons of data at first year

Using baseline-adjusted data at one year, intergroup difference of studied indices was not statistically significant (**Tables 1**-**3**, all P>0.050). 

## Inter-group comparisons of changes (baseline−one year)

Mean improvement of 0.06±0.22 (22.2%) and 0.02±0.25 logMAR (7.9%) in mesopic UDVA and 0.01±0.13 (14.3%) and 0.07±0.13 logMAR (87.9%) in mesopic CDVA was observed in the 5- and 10-minute groups, respectively. Mean decline in scotopic UDVA was 0.01±0.16 (1.0%) and 0.03±0.17 logMAR (11.9%) and mean improvement in scotopic CDVA was 0.03±0.10 (35.5%) and 0.02±0.07 logMAR (22.2%), in the 5- and 10-minute groups, respectively. In 5- and 10-min group, the safety index for mesopic (1.06±0.03 vs. 09±0.11, P=0.774) and scotopic (1.09±0.24 vs. 0.99±0.12, P=0.347) CDVA was similar between the study groups. As demonstrated in **Table 1**, the change in all indices was not significantly different between the 5-min and 10-min groups (all P>0.050). Among CS variables, only changes in corrected contrast sensitivity 0.5, 1.1 and 2.2 were significantly different between the study groups (all P<0.050) (Tables 2,3). The linear regression analysis showed that these differences related to baseline values not CXL protocols; corrected contrast sensitivity 0.5 (Pgroup=0.261 and Pbaseline value<0.001), 1.1 (Pgroup=0.250 and Pbaseline value<0.001), and 2.2 (Pgroup=0.101 and Pbaseline value=0.054).

## Discussion

Keratoconus leads to irregular astigmatism, increasing aberrations, and consequently deteriorating visual acuity [**9**,**10**]. Under low light conditions, vision loss is exacerbated due to pupil dilation and increased aberrations [**11**]. Our result showed that keratoconic patients had better mesopic VA than scotopic VA. 

CXL strengthens the cornea by creating covalent cross-links in the stroma [**1**], and diminishes aberrations by reducing corneal irregularity [**12**]. In standard CXL, 3mW/ cm2 irradiance for 30 minutes (total dose 5.4 J/ cm) has shown acceptable safety [**13**] and efficacy [**1**] in photopic light condition. Regarding the efficacy of standard CXL in low light condition, it showed that the halo and night driving would be improved after treatment [**12**].

In accelerated methods, the equal dose principle is applied to decrease the procedure time by increasing the intensity and maintaining total power. In this field, type of protocols such as 9mW/ cm2+10min, 30mW/ cm2+3min, 18mW/ cm2+5min, and 45mW/ cm2+2min have been presented. Although theoretically these protocols have the same total dose, different clinical results have been reported. Studies indicate that the two 5- and 30-minute protocols achieve the same short-term results [**14**], but in medium-term (18 months), the 5-minute protocol is less effective in flattening the cornea compared to the standard method despite halting keratoconus progression [**3**].

In clinical decision-making, it is crucial to know how far the procedure time can be reduced without sacrificing efficacy. So, several studies have compared accelerated protocols. Better photopic CDVA at 1 year was reported with the 5-minute approach compared to the 10-minute method and even the 30-minute protocol [**5**]. Our study showed similar mesopic and scotopic UDVA and CDVA outcomes with the 5- and 10-minute protocols. These inter-study differences might be related to different lighting conditions or patients’ baseline visual status. A study [**5**] reported CDVA increased 0.10 decimal in the 5-minute group, and the improvement was about 0.01 and 0.03 logMAR (0.04 decimal) in both lighting conditions in our study. Baseline CDVA was 0.2 in their study and 0.8 decimal (0.14 and 0.10 logMAR in each protocol) in ours. Thus, it could be said that cases with worse baseline vision gain more improvement [**15**]. In the study by Shetty et al. [**5**], enhancement of UDVA was 0.06 decimal in the 10-minute group, and in our study, it was 0.11 and 0.05 decimal in mesopic and scotopic conditions, respectively. This dissimilarity is clinically negligible.

As presented in the results, two CXL protocols decreased corneal HOAs similarly. In a 1-year study of 10-minute CXL, coma, trefoil, SA and total HOA indices reduced from 0.01 to 0.04 [**16**], and in another report [**17**], changes between 0.07 decrease to 0.07 increase were reported after 30-minute CXL. Although the mean decrease in our study was considerably greater compared to the two above studies, the variance of changes was larger as well. The difference observed in our results could be related to pupil dilation in our study under low light condition or the repeatability of different measurement devices in types of severity of keratoconus. We previously showed that OPD Scan III has lower repeatability for measurement of HOAs in cases with Kmax more than 53.0D [**18**]. 

Also, we showed that the improvement of lower spatial frequencies is related to baseline values and not to CXL [**19**]. In this report, linear regression model appeared that baseline values lead to CS improvement not CXL protocols. 

We compared changes in corneal biomechanics after these protocols [**20**] and showed the better improvement by 5-min method. The effect of CXL protocols may appear before visual function. Finally, it seems that 5- and 10-minute CXL protocols improve vision, HOAs and contrast sensitivity in low light conditions similarly. 

**Acknowledgment**

None.

**Funding**

None.

**Conflict of interest**

All authors certify that they have no affiliations with or involvement in any organization or entity with any financial interest or non-financial interest in the subject matter or materials discussed in this manuscript.
